# Interventions against Social Isolation of Older Adults: A Systematic Review of Existing Literature and Interventions

**DOI:** 10.3390/geriatrics6030082

**Published:** 2021-08-25

**Authors:** Jaya Manjunath, Nandita Manoj, Tania Alchalabi

**Affiliations:** Department of Geriatrics & Palliative Medicine, The George Washington University School of Medicine and Health Sciences, Washington, DC 20052, USA; jayamanjunath@gwmail.gwu.edu (J.M.); nandita@seniorswithskills.org (N.M.)

**Keywords:** social isolation, loneliness, interventions

## Abstract

Social isolation is widespread among older adults, especially those confined to living in nursing homes and long-term care facilities. We completed a systematic review evaluating the effectiveness of 20 interventions used to combat social isolation in older adults. A scoring mechanism based on the Joanna Briggs Appraisal Checklist was utilized to determine the quality of the studies. Searches were conducted in “MedLine”, “PubMed”, “PsycINFO” and “Aging and Mental Health”. Studies completed on group and person-centered interventions against social isolation were the highest quality as the social isolation experienced by older adults decreased after the intervention, and this effect continued in follow-up studies. Other interventions such as volunteering-based interventions also alleviated isolation; however, follow-up studies were not completed to determine long-term efficacy. Given the increase in social isolation faced by older persons during the pandemic, our review can be utilized to create effective interventions to reduce social isolation.

## 1. Introduction

Social isolation and loneliness are widespread among older adults, especially for those currently living in nursing homes and long-term care facilities [1]. As the population of older adults is increasing, social isolation has become more prevalent, affecting the mental, physical and social health of older persons. Loneliness and social isolation of older adults are considered an international public health issue [2], affecting millions of adults above the age of 65. The term “social isolation” is defined as a limited amount of social interaction, relationships, connections and social support [3].

The direct health outcomes of social isolation include an increased risk of premature death, a 50% increased risk of dementia, a 29% increased risk of heart disease and a 32% increased risk of stroke [4]. In addition to the physiological health consequences, previous studies have found associations between social isolation and behavioral and psychological problems [5].

There is a direct correlation between a lack of participation in social activities and cognitive decline among older persons. A 22-year follow-up study concluded that older adults living in a nursing home presented a greater cognitive decline than older adults living at home [6]. In addition, one study found that there was a 30% increase in the chance of developing cognitive impairments in those that reported loneliness [7]. This further reinforced the idea that loneliness and isolation contribute to the cognitive decline reported in older adults. A number of interventions combating social isolation have been implemented and analyzed. Volunteering programs, psychosocial group interventions, telephone calls and friendship enrichment clubs are examples of interventions seen in the literature. While many of these interventions show improved scores in loneliness- and social-isolation-related measures, the positive effects of these results are mainly short term. Hence, there is a need to develop feasible long-term interventions against social isolation tailored to older adults living at home, and in nursing homes.

As we progress in the COVID-19 pandemic, the issue of social isolation among all populations, particularly older persons, continues to grow. These older adults are at a higher risk of suffering negative consequences, and hence are asked to practice rigorous social distancing measures [8]. In addition to facing cognitive decline, declining physical health among older persons as a result of reduced physical activity has been reported amid COVID-19 [8]. Thus, the development of highly effective short-term and long-term interventions against social isolation are applicable to older adults.

In this systematic review, we present 20 studies of social isolation interventions and evaluate the effectiveness of the various interventions. Our objective was to assess the quality of the studies and to identify effective recommendations for interventions against social isolation.

## 2. Materials and Methods

### 2.1. Study Search

**Figure 1:**
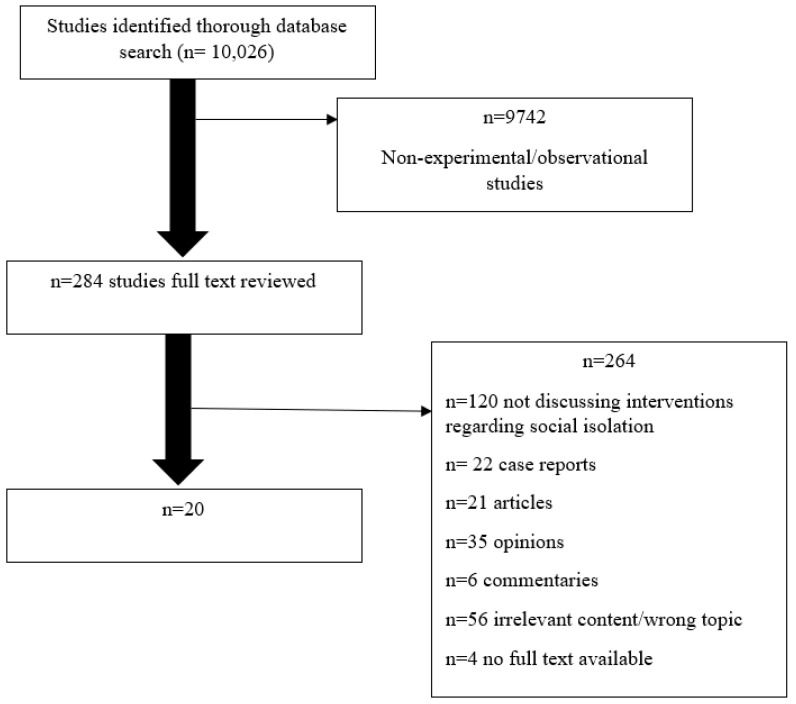
Process used to obtain final articles for analysis.

This review began with searches conducted in “MedLine”, “PubMed”, “PsycINFO” and “Aging and Mental Health” databases and journals to conduct a systematic review. The following key words were used with no date restrictions: “Social Isolation” or “Alone” or “Loneliness” and “Older persons” or “Older adults” or “Retired” and “Interventions” or “experiment” or “Social Interaction” or “Program” or “Social” or “Support” or “self-esteem”. The results demonstrate an abundance of literature pertaining to the negative consequences of isolation in the older population, but it was clear that research was lacking in the area of interventions to combat social isolation in older adults. A total of 10,026 articles were obtained from a mass search of databases, and a reviewer screened titles and abstracts (Figure 1). Out of the 10,026 articles after screening, 9743 articles did not meet the criteria to be selected, as they were not observational or experimental studies, and did not address isolation in older adults. From the remaining 283, the inclusion criteria were applied and a total of 20 complete articles were extracted for analysis.

### 2.2. Study Selection

Eligible studies met the following criteria:

- The study was related to older adults above the age of 50 in some way.
- The interventions were targeted towards older adults experiencing loneliness, and a method was proposed to combat isolation.
- The studies recorded an outcome from participants in a study addressing interventions to alleviate social isolation, and outcomes were reported to analyze treatment impacts
- The articles were published in English
- The articles consisted of quasi-experimental, observational or randomized clinical trial studies.

To address the effectiveness of interventions, experimental interventions and observational (qualitative) and quasi-experimental studies were selected. The interventions were divided into the following categories:

- Community-based approach;
- Psychosocial groups/rehab;
- Friendship enrichment clubs;
- Experimental study on social isolation and interventions;
- Early retirement;
- One-on-one interventions;
- Volunteering.

### 2.3. Quality Assessment of Studies Included

The Joanna Briggs Appraisal Checklist [9] was used for randomized clinical trials, qualitative research and quasi-experimental studies. The checklist for randomized controlled trials consisted of 13 criteria that were graded based on whether or not the study met them (“yes”, “no”, “unsure”, “NA”). The quality of the randomized controlled trials was assessed using this checklist. The Joanna Briggs Checklist was also provided for quasi-experimental studies, and this contained 9 sections that were used to evaluate the article quality. The JBI scores are provided for each study in Table 1.

## 3. Results

The 7 observational and 13 experimental intervention studies selected for this systematic review were classified into five different types of interventions (Supplemental Table S1). The classifications included volunteering-based interventions, group interventions, friendship-centered interventions, person-centered interventions and health-promoting interventions. Most studies we found were completed over a time frame of 1–12 months, had a sample size *n* < 100 and were either group interventions or friendship-centered interventions.

Furthermore, the effectiveness of these interventions on social isolation or loneliness, overall health and life satisfaction was determined (Table 1). The analysis of various interventions showed benefits to overall life satisfaction, but many failed to show significant long-term effects, unless interventions were continued. For example, studies conducted by [10,11] were shown to have beneficial effects on alleviating social isolation, but the effectiveness in the long run could not be concluded due to a lack of long-term follow-up studies conducted. This was explicitly mentioned in the limitations of the studies, implying that a lack of long-term evidence is a gap in the literature regarding effective interventions for social isolation. Community-based interventions and group interventions were shown to be good candidates, as they improved the overall satisfaction and health of the older adults.

Scores were assessed for each of the interventions analyzed, based on the Joanna Briggs Appraisal checklist. Based on this checklist and the overall efficacy of interventions, group interventions, which consisted of community involvement and the sharing of personal experiences, and person-centered interventions were found to be the most beneficial, especially when carried out for long periods of time. Specifically, the group intervention carried out by [12] and the person-centered intervention carried out by [13] showed significant mental and physical benefits while alleviating social isolation in older adults. These interventions addressed long-term implications and alleviated isolation in older adults. Specifically, the person-centered approach was also effective as it addressed the benefits of overall life satisfaction and perceived control on alleviating loneliness. Additionally, group-based interventions are concluded to be effective, as they provide educational, cognitive and social support by providing older adults with networking opportunities.

**Table 1:** Effectiveness and Implications of interventions against social isolation.

Intervention Authors, Year	JBI Score	Intervention Effect on Social Isolation/Loneliness Score	Overall Health/Life Satisfaction	Long Term Effectiveness	Implications
**Volunteering**					
[14]	4	Volunteer increases happiness	Volunteering increase this in men.	Religious volunteering positively impacts female happiness and male life satisfaction.	Gender may affect the effectiveness of volunteering
[15]	9	N/A	Volunteering decreased cognitive issues and dementia treatment likelihood	Consistent volunteer work is an effective intervention	Intervention effective if consistent
**Group Interventions**					
[16]	5	Life satisfaction, showed insignificant increase	Insignificant increase in overall mental and life satisfaction	Network building showed insignificant increases in quality of life, health.	Insignificant differences, so network building moderate effect.
[17]	5	Loneliness and isolation scores insignificantly lower than pretest.	Insignificant increases in total support satisfaction, positive affect and decreases in total support needed, and negative affect.	No significant long-term differences.	Insignificant effects in alleviating loneliness.
[18]	6	Loneliness alleviated and persisted 3 months later.	Groups socially activated participants.	Experiencing things together promoted the sharing of feelings.	Group intervention is effective.
[19]	3	Improvements shown in mastery, stress and loneliness	Improvements shown in stress, so overall health quality better.	Participants with highest education, significant difference from all but low level.	Community based intervention promote health and independence.
[20]	9	Meetings positively impact the social support	No improvements shown	No effect on other aspects of social support/loneliness.	Helps with social support but not isolation.
[21]	5	Social activation increased in the experimental group	Plasma level of testosterone, dehydroepiandosterone & estradiol increased & Hemoglobin A1C decreased.	Larger increase in the first 3 months, but still shows positive changes in 6 months.	Potentially effective program to reduce isolation but can have physiological effects.
[22]	6	Significantly lower loneliness and higher number of confidants and satisfaction scores	No measures of overall health, but more confidants and satisfaction in experimental groups.	The experimental group had impacts on alleviating loneliness.	This psychosocial group intervention is successful
[23]	9	Group without intervention experienced a decrease in perceived social support and an increase in perceived loneliness.	No implications on overall health.	Intervention did not improve loneliness in experimental group.	Not most effective intervention, but may increase cognitive functioning, and decrease depression
[10]	7	Improvement in well being lasts at least 3 months	Improvement in health and life satisfaction	Need to study long term effects, no data to support it.	Seems beneficial intervention, but need long-term studies
[12]	7	Participants gained social support	Mean subjective well-being scores higher for intervention group	Loneliness scale score decreased after the program and 6 months later	Community based programs allowing shared experiences have potential for success
**Friendship Centred Interventions**					
[24]	7	Older people value being in a community and independence to make connections.	Improved well-being, social Relations, mental/physical health	Friendship clubs address all areas, but need more research	Friendship clubs beneficial, but observational, so researcher influence
[25]	6	Overall alleviates immediate loneliness, but no decline over course.	Social and emotional loneliness, declined over study course	Immediate benefit but no loneliness decline long term.	Overall, help alleviate loneliness, no direct decline.
**Person Centred/One-on-One Intervention**					
[13]	7	Associated with feeling of control related to loneliness – older adults with more control may be able to cope with social isolation.	Life satisfaction is related to the psychosocial needs of residents	May have long term effects if given control and choice over their schedule.	Fulfilling preferences are an appropriate intervention for social isolation.
[26]	7	Some improvement in mental health scores, statistically insignificant.	Some form of improvement in mental health.	No statistical difference between intervention and control groups.	Peer telephone dyads were not an effective intervention
[11]	6	Participants receiving a friendly visitor showed a statistically significant difference in satisfaction	Clinical improvements occurred in the level of health.	Extensive research needed to verify program effectiveness.	Seems effective intervention but need long term research.
[27]	8	Significantly better health after visits only in the subgroup with poor health at baseline.	Benefits can be gained from home visits if health problems already present	Need long term research; effective for those with pre-existing conditions.	Only beneficial to those with pre-existing problems, can’t generalize.
**Health Promoting/Social Support Interventions**					
[28]	9	Positive effect of health-promoting interventions on older adults’ lifestyle.	Significant difference in total average scores of lifestyle between intervention and control groups.	Improvement in lifestyle conditions predicted to be long term but need to investigate further.	Beneficial intervention to improve lifestyle of older adults
[29]	6	Significantly less loneliness and more social support and well-being at 6 months, but no statistically significant difference at 12 months	Increase in computer comfort, efficacy and proficiency.	No statistically significant difference at 12 months, so long term effects minimal.	PRISM is a good tool for social connectivity but may only be a short term intervention.

## 4. Discussion

In this systematic review, we describe the current interventions against social isolation of older adult citizens. Overall, there are a wide range of interventions, including support groups, friendship programs, telephone calls and volunteering. These interventions have been shown to improve loneliness scores, stress, overall quality of life and mental health. Based on our review, recommendations can be made for older adults to participate in these programs on a regular, long-term basis. However, there is still a need for interventions to be developed that increase the individual independence of older persons.

After analyzing various types of interventions, we concluded that group interventions and person-centered interventions were the most effective programs, especially when carried out for long periods of time. Specifically, group interventions carried out in [12,18,19,20,21,22] showed beneficial long-term outcomes in alleviating social isolation. Group interventions resulted in better mental and physical health outcomes as well as a lower level of loneliness. Additionally, there was an increased level of perceived social support and social activation after group interventions were implemented to alleviate isolation. These specific studies conducted long-term follow ups to further reinforce the effectiveness of these interventions. Next, person-centered interventions carried out by [13] showed significant mental and physical benefits, while alleviating social isolation in older adults. The person-centered approach is known to be directed towards the client in an empathetic, nondirective manner. It is known to empower and motivate clients by recognizing their individuality [30]. In this approach, older adults felt capable of controlling their loneliness, resulting in reduced scores of social isolation. Overall, a higher perceived control over isolation levels and increased social activation through long-term interventions were shown to lead to reduced feelings of social isolation in older adults.

Although these interventions were concluded to be effective due to their long-term implications, we further assessed them based on the Joanna Briggs Appraisal Checklist. This checklist was used to assess the quality of the study depending on the type of study conducted. High Joanna Briggs scores (7/10) were found in a group intervention by [12], and a person-centered intervention by [13]. This finding allowed us to reinforce the conclusion that person-centered and group interventions are the most effective in alleviating social isolation. Since a higher score corresponds to a higher quality of results, these articles demonstrated the most relevant results regarding effectiveness in combatting isolation in older adults.

In order to implement successful interventions to combat isolation, the long-term efficacy of these interventions must be studied. In the following studies, [10,11,27], long-term follow-up data were not available. These studies have shown potential short-term benefits in terms of alleviating loneliness; however, long-term studies are necessary to confirm the effects on the older adult population. Given that social isolation is a prevalent long-term issue faced by older adults, interventions implemented against loneliness must have proven long-term efficacy.

The lack of studies conducted on previously implemented interventions against social isolation, combined with limited data on the long-term effectiveness of these interventions, makes it difficult to ascertain the efficacy of these interventions. Furthermore, many of the studies chose samples restricted to a specific area and did not control for confounding factors such as gender and socioeconomic status, thus affecting the quality of the results. Additionally, most of the experiments did not employ blinding methods, which may have impacted the participants’ perception of the intervention.

The unfolding of COVID-19 has led to many older adults facing harsh and grueling conditions at long-term care facilities and nursing homes. Since COVID-19, older adults have been unable to participate in group face-to-face activities or programs. Several nonprofit organizations have started “Online Buddy Programs” and groups have completed telephone chats with older adults [31]; however, many of these services are inaccessible to older adults due to a technology gap. Additionally, statistical evidence in this area is still inadequate to conclude significant benefits regarding social isolation. To increase independence among older adults while combatting social isolation, we recommend that older adults develop virtual connections, and become volunteers in their community. Developing innovative and accessible technology for older adults, such as person-centered applications, will be beneficial to allow older adults to maintain communication between one another and their families [4]. The importance of developing community programs to combat social isolation during COVID-19 cannot be understated.

In conclusion, studies completed on group and person-centered interventions were the highest quality as the social isolation experienced by older adults decreased after the intervention, and this effect continued in follow-up studies. Our results can be used to guide effective interventions to alleviate loneliness in older adults, as we recommend the development of group/person-centered interventions in nursing homes and long-term care homes. Additional research studies are warranted to understand the long-term efficacy of these interventions on the prevention or reduction of social isolation in elders.

## Data Availability

Not applicable.

## Supplementary Materials

The following are available online at https://www.mdpi.com/article/10.3390/geriatrics6030082/s1.
