# Supplementary material for: Interventions against Social Isolation of Older Adults: A Systematic Review of Existing Literature and Interventions
Source: Geriatrics (Basel). 2021 Aug 25;6(3):82. doi: 10.3390/geriatrics6030082 (PMC8395923; doi:10.3390/geriatrics6030082)
Supplement: Supplementary file 1 [file geriatrics-06-00082-s001.zip › geriatrics-1322022-supplementary.pdf]

Supplemental Table 1. Study intervention demographics. *

Intervention	Location of Study	Study Length	Sample
**Volunteering**			
[14]	Chile, Mexico and Spain	1994–1998, 2005–2009, 2010–2014	n = 1,699, ages 61–80
[15]	Swedish National Prescribed Drug Register	2010, 2012, 2014	n = 531, no volunteering<br>1) n = 220, discontinuous volunteering<br>2) n = 250, continuous volunteering
**Group Interventions**			
[16]	Chicago	3 months	n = 26, network-building visiting program<br>1) n = 26, relationship-oriented visiting program<br>2) n = 13, control group
[17]	Canada	20 weeks	n = 28, widowed older adults, age >55, 4 face to face support groups
**Psychosocial Group Intervention** [18]	Finland	3 months	n = 117, 3 groups: art and inspiring activities, group exercise and discussions, therapeutic writing and group therapy
**Community-Based Approach** [19]	Las Vegas, Nevada and Rural Clark County	4 months	n = 339
**Community Approach** [20]	Sweden	1 year	n = 416, ages 70+<br>1) n = 227: intervention group<br>2) n = 189: control group
**Social Activation Program** [21]	Stockholm, Sweden		n = 60<br>1) n = 30: experimental group<br>2) n = 30: control group
**Psychosocial Group Intervention** [22]	Japan	6 weeks	n = 50, age < 65<br>1) n = 25: experimental<br>2) n = 25 control
**Cognitive Enhancement Program, Group Intervention** [23]	USA	3 months	n = 58, ages 61–98 years old<br>1) n = 29: experimental group<br>2) n = 29: control
**Intervention Program** [10]	Sweden	24 weeks	n = 65 (random allocation to experimental and control groups)
**Social Isolation Prevention Program** [12]	Tokyo, Japan	6 months	n = 63<br>1) n =21: intervention group<br>2) n = 42: control group
**Friendship-Centered Interventions**			
**Friendship Clubs** [24]	UK	3 years	n = 100
**Individual Friendship Enrichment Program** [25]	Dutch	6 weeks	n = 239
**Person Centered/One-on-One Intervention**			

[13]	Louisville, KY, USA		n = 65, ages 51–90
**Peer Telephone Dyads** [26]	USA	10 weeks of staff telephone calls then pairing in dyads	n = 265
**Individual Visits** [11]	Southern Ontario, Canada	6 weeks	n = 26
**One-on-One Visits** [27]	The Netherlands	3 years	n = 580<br>1) n = 292: experimental group<br>2) n = 288: control group
**Health-Promoting/Social Support Interventions**			
**Health-Promoting Interventions** [28]	Tehran, Iran	12 months	n = 464<br>1) n = 232: control group<br>2) n = 232: experimental group
**PRISM System: A Specially Designed Computer System for Older Adults (Randomized Trial)** [29]	USA	Collection at 6 and 12 months	n = 300

*Same reference as the main text.
